# Genome-Wide Association Study of Salt Tolerance-Related Traits during Germination and Seedling Development in an *Intermedium-Spike* Barley Collection

**DOI:** 10.3390/ijms231911060

**Published:** 2022-09-21

**Authors:** Mohammed A. Sayed, Andreas Maurer, Thomas Schmutzer, Thorsten Schnurbusch, Andreas Börner, Mats Hansson, Klaus Pillen, Helmy M. Youssef

**Affiliations:** 1 Leibniz Institute of Plant Genetics and Crop Plant Research (IPK), Corrensstr. 3, OT Gatersleben, 06466 Seeland, Germany; 2 Faculty of Agriculture, Assuit University, Assuit 71526, Egypt; 3 Institute of Agricultural and Nutritional Sciences, Faculty of Natural Sciences III, Martin Luther University Halle-Wittenberg, 06120 Halle, Germany; 4 Department of Biology, Lund University, Sölvegatan 35B, 22362 Lund, Sweden; 5 Faculty of Agriculture, Cairo University, Giza 12613, Egypt

**Keywords:** barley, salinity, GWAS, candidate genes, seedling development, germination

## Abstract

Increased salinity is one of the major consequences of climatic change affecting global crop production. The early stages in the barley (*Hordeum vulgare* L.) life cycle are considered the most critical phases due to their contributions to final crop yield. Particularly, the germination and seedling development are sensitive to numerous environmental stresses, especially soil salinity. In this study, we aimed to identify SNP markers linked with germination and seedling development at 150 mM NaCl as a salinity treatment. We performed a genome-wide association study (GWAS) using a panel of 208 *intermedium*-spike barley (*H. vulgare* convar. *intermedium* (Körn.) Mansf.) accessions and their genotype data (i.e., 10,323 SNPs) using the genome reference sequence of “Morex”. The phenotypic results showed that the 150 mM NaCl salinity treatment significantly reduced all recorded germination and seedling-related traits compared to the control treatment. Furthermore, six accessions (HOR 11747, HOR 11718, HOR 11640, HOR 11256, HOR 11275 and HOR 11291) were identified as the most salinity tolerant from the *intermedium*-spike barley collection. GWAS analysis indicated that a total of 38 highly significantly associated SNP markers under control and/or salinity traits were identified. Of these, two SNP markers on chromosome (chr) 1H, two on chr 3H, and one on chr 4H were significantly linked to seedling fresh and dry weight under salinity stress treatment. In addition, two SNP markers on chr 7H were also significantly associated with seedling fresh and dry weight but under control condition. Under salinity stress, one SNP marker on chr 1H, 5H and 7H were detected for more than one phenotypic trait. We found that in most of the accessions exhibiting the highest salinity tolerance, most of the salinity-related QTLs were presented. These results form the basis for detailed studies, leading to improved salt tolerance breeding programs in barley.

## 1. Introduction

Abiotic stresses such as salinity, drought, low or high temperatures, floods and frost are global environmental phenomena that negatively affect many plant species and are expected to become more severe and widespread [1]. Soil salinity problems exist in more than 100 countries, and their global area is approximately 1.13 billion hectares. In total, 20% of the worldwide cultivated land and 33% of the irrigated land are salt-affected [2,3,4]. The salinity stress significantly reduces plant growth and negatively affects photosynthesis, consequently resulting in biomass and yield reduction [5]. The seed germination and seedling establishment processes are the most critical stages in the plant life cycle that contribute substantially to final yield. Seed germination and seedling emergence take place in the soil rhizosphere where there is plenty of salt accumulation, and thus, these two stages are most sensitive to soil salinity stress [6].

Seed germination and plant growth and development interfere with salinity through (i) essential nutrients uptake imbalance, (ii) osmotic pressure imbalance, i.e., initiating water shortage, (iii) ion toxicity and (iv) production of reactive oxygen species (ROS) that act at the cellular or at whole plant level to cause physiological and biochemical defects, resulting in reduced germination, suppressed seedling growth and poor harvest [7]. The osmotic adjustment may be triggered first under high-saline conditions [8], and then, plants regulate cellular ion homeostasis by the actions of multiple transporters in response to ion toxicity [9,10], while osmolyte adjustment is a key element under salinity and drought stresses [11]. It is known that several stress-regulated transcription factors are activated in response to both salt and drought stresses, such as WRKY transcription factors [12], Apetala 2 (AP2) [13], Basic leucine zipper (bZIP) [14], and MYB [15].

Barley (*Hordeum vulgare* L.) is among the most important cereal crops worldwide and has been known to be cultivated for about 10,000 years. Naturally, it shows high levels of drought and salinity tolerance such that it has the advantage of growing in marginal environments that are unsuitable for other cereal crops [6,16,17,18] Therefore, it is usually used as a model crop for understanding mechanisms of germination and seedling and plant development in monocots under salinity stress [19,20]. However, salinity causes a significant reduction in barley growth and seed yield. The level of salinity tolerance of the barley genotype depends on the ability to develop and survive under salinity stress [21,22]. Salt stress on barley seed germination and seedling development has been examined in a number of studies to determine how barley responds to salinity and to develop new salt-tolerant lines to be used in breeding programs [6,23]. An increase in salinity levels in barley cultivation resulted in a decrease in germination rate, shoot length, root length, fresh and dry roots weights, and relative growth rate [24].

It is well known that salinity tolerance is controlled by several loci [25]. To reveal and clarify the genetic basis of this complex agronomic trait, genome-wide association studies (GWAS) have been gradually used. In rice, GWAS was executed to identify loci regulating salinity tolerance. GWAS identified new quantitative trait loci (QTL) on chromosomes (chr) 4, 6, and 7 that control salinity tolerance at the seedling stage [26]. In barley, at the germination stage, Mano and Takeda [25] stated 17 QTL controlling abscisic acid (ABA) responses on chr 1H, chr 2H, chr 3H and chr 5H, where the loci located on chr 5H were closely linked to salinity tolerance. Witzel et al. [27] studied QTL mapping using the Oregon Wolfe Barley population and found several regions on chr 2H, 5H and 7H that were associated with salt stress response at the germination stage. Thus far, little is known about the genes and genetic mechanisms associated with the identified QTL for salinity tolerance at germination [28]. The current developments in GWAS technology allow for the genotyping of thousands of gene loci across hundreds of accessions using high-throughput markers to enhance breeding efficiency [29]. GWAS can locate polymorphisms and underlying genetic loci accounting for phenotypic variations [30,31].

To develop new salt-tolerant barley lines to be used in breeding programs, there needs to be screening of the available barley genetic resources and worldwide collections under salinity stress conditions to select the best lines for this goal. To the best of our knowledge, among QTLs governing response to salinity stress tolerance in barley, only a few have been identified at germination and seedling levels. In this study, we screened a collection of 208 worldwide barley collections, so-called *intermedium*-spike accessions as described by Youssef et al. [32,33] at germination and seedling stages and used GWAS analysis to identify salinity tolerance loci associated with salt tolerance-related traits at germination and seedling stages in this collection. We tested the collection under control and salinity treatment (150 mM NaCl). The germination and seedling growth parameters of these accessions were assessed, and stress tolerance indices were calculated. The GWAS analysis of the tested traits associated with salinity tolerance at germination and seedling stages was conducted using 10,323 single nucleotide polymorphism (SNP) markers. At the germination and seedling stages, we identified 38 putative QTL and 153 HC predictive genes associated with salt-tolerance traits that can be used in future barley breeding resilience programs.

## 2. Results

### 2.1. Trait Variability and Heritability Estimates of Intermedium-Spike Barley Population

The analysis of variance for germination and seedling growth parameters revealed highly significant differences among the *intermedium-spike* barley accessions under control and 150 mM NaCl conditions (Table 1). In addition, the combined ANOVA for all studied parameters revealed highly significant differences among accessions, between treatments and for their interaction as well. Moreover, the coefficient of variation (CV) values were higher under salinity conditions in the majority of traits studied as compared to the control. Wide ranges (minimum and maximum values) of the studied traits were observed under both treatments (Table 2). These results indicate sufficient variability and different responses to salinity stress that exist in the *intermedium-spike* barley material.

Moderate to high values of coefficient of determination (R^2^) and heritability (H_b_) estimates were noted under both conditions. Under control, the highest R^2^ and H_b_ were observed for germination index (GI) (0.95 and 97.6%), while the lowest ones were recorded for water content percentage (WCP) (0.78 and 85.7%). Under salinity, shoot length (ShL) exhibited the highest value of R^2^ (0.91) and H_b_ (95.1%), whereas seedling fresh weight (SFW) had the lowest values of R^2^ (0.61) and H_b_ (67.8%).

### 2.2. Means of the Studied Traits and Response to Salinity

The relative salinity performance (R%) on the investigated parameters ranged between −42.07% for seedling vigor index (SVI) and 24.86% for seedling dry weight (SDW) (Table 2). It could be observed that the majority of the parameters were reduced under salinity conditions, while mean germination time (MGT), root:shoot ratio (RSR) and SDW were increased.

Among the 208 accessions tested, 22 of them including HOR 11747, HOR 11718, and HOR 11640 showed 100% germination under salinity conditions. These were the fastest germination accessions, but they recorded the lowest values of MGT with an average of 2.5 days (Table S1). The accessions HOR 5837, HOR 5017, HOR 11256 and HOR 11275 showed the tallest shoots under 150 mM NaCl conditions, while accessions HOR 11291, HOR 11275, HOR 11256, and HOR 6661 recorded the tallest root length (Table S1). Accessions HOR 11291, BCC 747, HOR 11275, and HOR 11256 had the highest WCP under 150 mM NaCl treatments. By these criteria, these accessions could be characterized as salt-tolerant genotypes. Together, we found HOR 11747, HOR 11718, HOR 11640, HOR 11256, HOR 11275 and HOR 11291 to be the most salinity-tolerant accessions in the *intermedium*-spike barley collection.

### 2.3. Phenotypic Correlation among Germination and Seedling Growth Parameters

**Figure 1:**
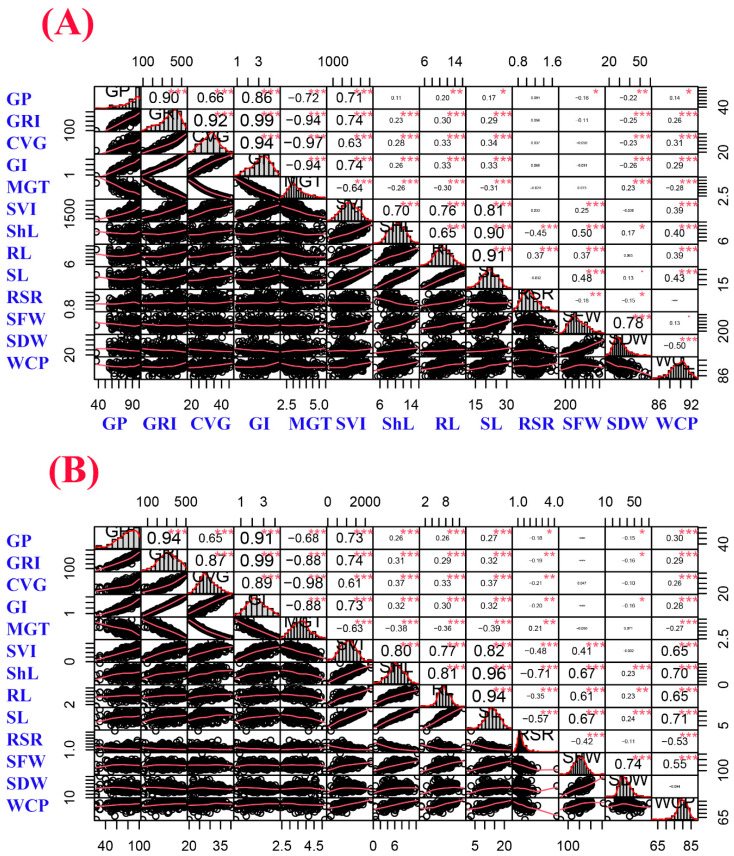
Correlation matrix of germination and seedling growth parameters under (**A**) control and (**B**) salinity conditions (150 mM NaCl). The distribution of each trait is shown on the diagonal. The bivariate scatter plots with a fitted line are displayed on the bottom of the diagonal, and the correlation coefficients plus the significance level as stars are shown on the top of the diagonal. *, **, *** are significant, highly significant, and very highly significant at 0.1, 0.05, 0.01, and 0.001 probability level, respectively. Trait abbreviations are given in Table 1 footnote.

Pearson correlation coefficients were calculated among germination and seedling growth parameters under control and salinity conditions (150 mM NaCl), which are displayed in Figure 1. Under both treatments, the *intermedium-spike* barley population (208 accessions) displayed a normal distribution for the majority of the studied traits as shown in Figure 1. Strong, positive and highly significant correlations were observed between germination percentage (GP) and each germination rate index (GRI), coefficient of velocity of germination (CVG), GI and SVI under both treatments. MGT was negatively and highly significantly correlated with GP, GRI, CVG and GI under both treatments. Correlation coefficients among germination growth parameters were much higher under control compared to salinity. Furthermore, ShL showed highly significant and positive correlations with SVI, root length (RL), seedling length (SL), SFW and WCP under both treatments. In addition, it could be observed that correlation coefficients among seedling growth parameters were much higher under salinity stress compared to control.

### 2.4. Salinity Tolerance Indices (STI) and Their Relationships

**Figure 2:**
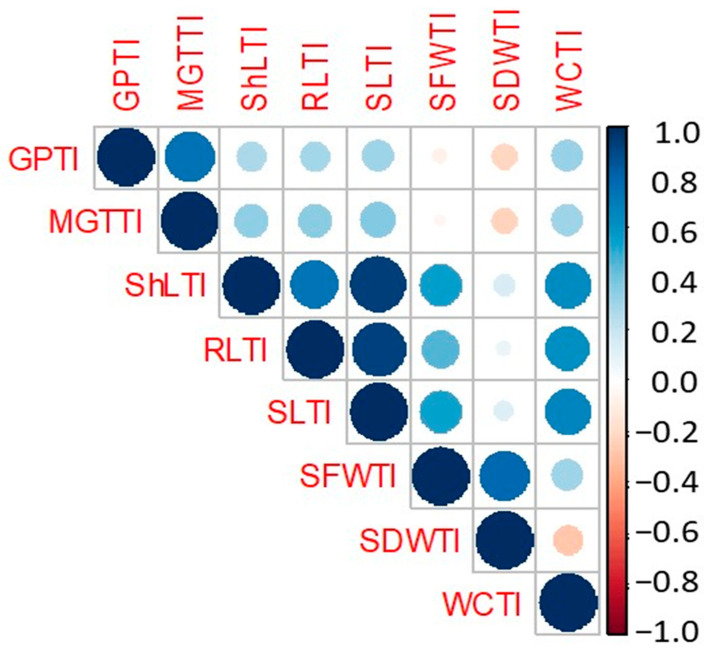
Pearson correlation matrix among salinity tolerance indices (STI). Trait abbreviations are given in Table 3 footnote.

Eight salinity tolerance indices related to GP, MGT, ShL, RL, SL, SFW, SDW, and WCP were subjected to the analysis of variance and correlation and are displayed in Table 3 and Figure 2. The analysis of variance revealed highly significant differences among the *intermedium-spike* barley accessions for all tested salinity indices. High coefficients of determination (R^2^) coupled with high estimates of broad-sense heritability (H_b_) were obtained for the salinity indices. They ranged between 0.77 and 85.2% seedling fresh weight tolerance index (SFWTI) and 0.92 and 95.9% mean germination time tolerance index (MGTTI) for R^2^ and H_b_, respectively. The seedling dry weight tolerance index (SDWTI) exhibited the highest mean (1.29) among salinity tolerance indices, while shoot length tolerance index (ShLTI) showed the lowest value (0.65). The minimum and maximum values of the salinity tolerance indices are shown in Table 3. For each line, the mean and summary statistics of the tested indices are displayed in the supplementary Table S2. According to the germination tolerance index (GPTI), the accessions HOR 11275, HOR 11618, HOR 11640 and HOR 11747 exhibited the highest values of GPTI with an average of 1.3. High values of STI indicate that these accessions are tolerant to the stress. For MGTTI, the accessions HOR 11633, HOR 11618, HOR 11718, and HOR 7076 had the highest values of MGTTI and recorded 1.69, 1.66, 1.63 and 1.58, respectively. These accessions completed their germination in a short time and could be considered tolerant genotypes to salinity stress at the germination stage. For the seedling length tolerance index (SLTI), the accessions HOR 7113, HOR 11256, HOR 11291, HOR 5772, and HOR 11275 showed the highest values of the seedling length tolerance index with an average of 1.34 and were considered the tallest seedlings under salinity conditions compared to control. For the water content tolerance index (WCTI), the accessions HOR 11256, HOR 11291 and HOR 12354 had the highest values of WCTI with an average of 0.98, indicating a high water content in their tissues under salinity conditions near to their content under control. These STI results confirmed the superiority of HOR 11747, HOR 11718, HOR 11640, HOR 11256, HOR 11275 and HOR 11291 accessions in salinity tolerance in the *intermedium*-spike barley collection.

A Pearson correlation coefficients matrix was calculated among the eight salinity tolerance indices (Figure 2). GPTI was positively and highly significantly correlated with MGTTI, ShLTI, RLTI, and SLTI. In addition, WCTI was associated positively and highly significantly with all salinity tolerance indices except for SDWTI, which was negative.

### 2.5. QTL Detection

Both phenotypic and genotypic data were subjected to a QTL analysis to detect putative QTL associated with traits related to germination and seedling development under control and salinity stress and for eight salinity tolerance indices (Table S3). One QTL was identified for each of the traits GP, CVG, GI, MGT, SFW and SDW under control conditions and mapped on chr 1H, 2H, 6H and 7H. In addition, all these QTL showed positive additive effects for the major allele. In addition, thirteen QTL were detected for ten investigated traits out of thirteen under salinity conditions and mapped on chromosomes 1H, 2H, 3H, 5H and 7H. Six QTL regions were responsible for salinity tolerance in the *intermedium-spike* barley collection by increasing their performance mainly for traits CVG, SDW and GI (chr 1H), SVI (chr 5H), ShL (chr 2H) and SL (chr 7H), while the other detected QTL showed negative performance of the accessions for traits GP (Chr 5H), ShL (Chr 5H), SL (Chr 5H), SFW (Chr 1H and Chr 3H) and WCP (Chr 7H). Five QTL were identified for five salinity tolerance indices (ShLTI, RLTI, SLTI, SFWTI, and WCTI) out of eight. The QTL were located on chromosomes 2H and 7H, and the major SNP alleles showed negative estimates (Table 4). We found that the salinity-related QTL associated with marker 3_599466174, located on chr 3H was presented in all six accessions that showed superior salinity tolerance in this collection. The QTL associated with markers 1_546373322, 2_698868743, 6_7696839 and 7_629887676 located on chr 1H, 2H, 6H and 7H, respectively, were found in all six accessions except for HOR 11275.

### 2.6. Candidate Gene Prediction

Within the regions flanking each QTL, we searched for the high confidence (HC) genes from the barley reference genome assembly of Morex, version refseq 1 (Table 4 and Table S4) and its corresponding gene annotation [34]. We found 153 HC genes associated with 38 putative QTL. Of these, 22 SNPs were found inside possible HC candidate genes (Table 5), while the other 16 SNPs were located close to the HC genes. Based on their −log10 P values and % R^2^ values (Table 4), it may be possible that some of these candidate genes are involved in enhancing salinity tolerance at the germination and seedlings stages. For example, the candidate genes coding for the Cytochrome P450 superfamily protein, early nodulin-like protein, Phospholipase A1, Helicase-like protein, MYB domain protein, Homeobox-leucine zipper protein family and kinase family protein were found more than once, indicating a possible role in salinity tolerance during germination and seedling stages in barley. We found that the gene *HORVU4Hr1G085320* encoding a NAC domain protein was associated with SNP m4_631432489 and is proposed to regulate the SDW under salinity stress in barley. In addition, we found that duplication genes *HORVU1Hr1G090570* and *HORVU1Hr1G090580* both encode phospholipase A1-II 6 protein and are associated with marker 1_546373322. The QTL was associated with marker 3_599466174 presented in all six superior salinity tolerant accessions, encoding the NRT1/ PTR FAMILY 5.10 protein (Table 5 and Table S4).

## 3. Discussion

Salinity has a negative effect on many metabolic processes, causing a reduction in seed germination, seedling growth and plant development due to the effects of salt accumulation in the plant tissues [35,36]. Plant tolerance to salinity is a complex quantitative trait that depends on genetic and physiological factors, and any change induced by salinity is influenced by gene expression [37]. The genetic variation found in wild crop relatives shows a large amount of allelic variation in contrast to commercial elite cultivars [33]. This genetic variation can potentially contribute greatly to improvement of stress tolerance [38]. Among QTLs controlling response to osmotic stress [39,40], ionic stress [27,41] and salinity stress tolerance [28] in barley, only a few have been identified at germination and seedling stages. Therefore, our discovery of novel QTL associated with salinity tolerance traits in the worldwide *intermedium-spike* barley collection has the potential to improve crop salinity tolerance in barley breeding programs.

The results indicated significant negative effects of the salinity treatment for all seed germination and seedling growth related traits compared to control condition except for the MGT, RSR and SDW that increased under salinity stress. Despite that the accuracy of the results obtained might be due to the tight control of the experiment, these results are in contrast to many research studies that have stated that plant dry weight (DW) was reduced under salinity conditions in different crops including barley [6,42,43,44,45]. Our results were in agreement with what was found by Sayed et al. [46] studying the S42IL introgression library of 50 lines under salinity stress conditions and Angessa et al. [28] in the CM72 x Gairdner doubled haploid barley population. Additionally, Abdul Qados [47] and Allam et al. [48] reported that the fresh and dry matter production was found to increase in studied genotypes from lower to higher salinity levels. In these populations, seeds have been subjected to salt-induced physiological drought stress as a result of the high osmotic pressures generated by the salt environment, causing an observed reduction in germination and seedling growth traits. This process affects the ability of seeds to absorb water from the germination medium, consequently causing prolonged plant development or even preventing plants from absorbing water and consequently seedling and plant growth [49,50]. The *intermedium-spike* barley collection showed a wide range of salinity tolerance in all of the tested traits (Table 2). Across the population, the maximum reduction due to the salinity treatment was found in SVI by 42.07%, and the minimum reduction was found in WCP by 9.92%, while the maximum increase due to salinity treatment was found in SDW by 24.86%, and the minimum increase was found in MGT by 10.95% compared to the control condition. The salt-tolerance expressed in reducing the loss of water and increasing the seedling dry weight may be due to the increase in osmotic potential in seeds by nutrient uptake, which led to the absorption of more water under salinity stress [51]. The increase in RSR by 11.94% under salinity conditions may also be due to significantly reduced shoot dry matter production, shortened shoots, and elongated roots to obtain water [52]. Notably, where the SDW mean across all accessions under control condition was 30.0 g, 121 accessions showed SDW more than 35.0 g under salinity stress treatment; 77 of these accessions showed WCP >80%. These salt-tolerant accessions may be used in barley breeding programs for the improvement of salinity tolerance to develop environmentally smart cultivars to be used for climatic change scenarios. The STI recorded in this study showed high variation among the accessions. This finding was in agreement with Angessa et al., 2017 [28], and Allel et al., 2019 [53]. We suggested that it is better to use traits with high rates of variation such as STI (Table 3), WCP and SVI than the traits with low rates of variation to select accessions for salinity tolerance breeding programs [24,54]. The STI can be used as an indicator for selecting the salt-tolerance accessions from this collection.

The observed significant differences among the accessions and between the salinity treatments and control indicate various possible responses to salinity stress in this *intermedium-spike* barley collection. This variation among the accessions was reflected through the high estimates of broad-sense heritability under salinity conditions in the current study. The heritability estimates of the investigated traits were high (between 67.8 in SFW and 95.9 in MGTTI) for all traits under salinity treatment. These findings indicate that salinity tolerance related traits during germination and seedling development are genetically controlled to a high extend. Confirming our findings, high estimates of heritability in the same recorded traits at germination and seedling development under salinity stress were also obtained in other reports [6,42,44,46]. GWAS analysis makes use of historical recombination to identify regions of the genome that are responsive to traits using a high-resolution genome scan [55]. Several QTL have been reported for salt tolerance traits during germination and seedling development [28,46,56]. For example, QTL for salinity tolerance were identified on chromosomes 1H, 2H and 7H in the DH populations TX9425 × Franklin, YYXT × Franklin and in a worldwide collection of 206 barley accessions [57,58]. Some of those QTL were closely linked to significant markers reported in our study.

In this study, the GWAS analysis revealed that 55 significant SNPs, summarized to 38 putative QTL, may regulate salinity tolerance in the *intermedium-spike* barley collection. For the salinity-related traits as well as salinity tolerance indices, three QTL on chromosome 1H, four QTL on chromosome 2H, one QTL on chromosome 3H, four QTL on chromosome 5H, and three QTL on chromosome 7H were detected. These results confirmed that multiple small-effect genes act in combination to regulate salinity tolerance in barley during germination and seedling development [59]. Of the presented candidate gene families associated with these QTL, the kinase family protein and phospholipase A1 were also identified at germination and seedling stages in rice and barley under salinity stress [44,60]. The presence of phospholipase A1 in HOR 11747, HOR 11718, HOR 11640, HOR 11256 and HOR 11291 might explain their salinity-tolerant superiority since it aids in the biosynthesis of jasmonic acid, which is considered an important stress regulator [61,62]. Plants such as barley, cotton and wheat have been found to use the protein kinase superfamily genes to adapt to drought and salinity stress conditions [24,63,64,65]. In soybean plants with overexpressed protein kinases, the salt tolerance was significantly increased, suggesting that it may play a crucial role in salinity tolerance [66]. In *Arabidopsis thaliana*, the abscisic acid (ABA)-non-activated protein kinases regulate reactive oxygen species (ROS) homeostasis and trigger gene expression under salinity stress conditions [67]. The NAC domain transcription factor that we identified as a candidate gene associated with marker m4_631432489 enhanced the salinity tolerance in our barley collection, especially in two of the highest tolerant ones, HOR 11718 and HOR 11640. This result is in agreement with Li et al. [68] who found that salt stress influenced the expression level of GmNAC06 in soybean. NAC overexpression caused proline and glycine betaine accumulation in the cells that help to alleviate or avoid the negative effects of ROS. Similarly, it also regulates the Na+/K+ ratios to maintain ionic homeostasis in soybean hairy roots [68]. MYB transcription factors play an important role in abiotic stress responses in different plants such as *Arabidopsis* [69], peanut [70] and wheat [71]. In our study, we found that MYB domain transcription factors are associated with markers related to salinity tolerance. Through promoting expression of stress-associated genes and controlling osmotic and oxidizing substances, MYB domain transcription factors might help maintain cell homeostasis in response to drought and salt stress [72]. In transgenic *Arabidopsis* plants, salinity stress prompted lipase expression, enhancing salinity tolerance, which simplifies seed germination, vegetative growth, flowering, and seed set [73]. All of the above-mentioned gene families are found to be associated with stress tolerance in barley, including salinity, as reported in this study. Our results will form the basis for future studies to discover and verify the mechanism by which candidate genes play a role in salinity tolerance during germination and seedling development stages in barley. In this study, various promising barley accessions showed a high degree of salinity tolerance based on their STI, such as HOR 11747, HOR 11718, HOR 11640, HOR 11256, HOR 11275, and HOR 11291. These accessions could be used as the basis for our research plan to create improved salinity-tolerant barley population. Prior to this, we recommend greenhouses and field experiments in order to validate these results using a wider range of plant growth and yield traits under salinity stress conditions.

## 4. Materials and Methods

### 4.1. Plant Material and Genotyping

A set of 208 *intermedium-spike* spring barley accessions (*H. vulgare* L. convar. *intermedium* (Körn.) Mansf.) [74] of worldwide origin (Europe, East and West Asia, Africa and Americas) was used in this study. All information about this *intermedium-spike* barley accession collection and its genetic characterization has been published by Youssef et al. [33].

### 4.2. Experimental Design and Salinity Stress Treatments

The experiment was conducted at the Leibniz Institute of Plant Genetics and Crop Plant Research (IPK), Gatersleben, Germany, in a completely randomized design. The whole set of accessions was surface sterilized with 70% ethanol solution for one minute and rinsed with sterile distilled water several times, then briefly blotted. The seeds were placed on two layers of filter papers (C160; Ahlstrom-Munksjö, GmbH, Dettingen an der Erms, Germany) laying in crystal clear rectangular boxes (V3-92; Licefa GmbH & Co. KG, Bad Salzuflen, Germany). The salinity treatments, with three replicates, were conducted by watering the seeds with 150 mM NaCl (Sodium chloride CELLPURE^®^ ≥ 99.5 %, for cell culture and biochemistry, Carl Roth, GmbH, Karlsruhe, Germany), whereas deionized water was applied as a control treatment, and the seeds were placed in a versatile environmental test chamber (Model No. MLR-352-PE, Panasonic, Kadoma, Japan) for ten days, maintained at 20 ± 2 °C with 50 ± 5% humidity at 12 h light (200 μmol m^−2^ s^−1^) and 12 h dark periods per day. The seeds were considered germinated when the radicle reached at least 2 mm in length, and the number of the germinated seeds was counted daily after 24 h of incubation until the end of the experiment. 

### 4.3. Evaluation of Germination and Seedling Growth Parameters

Seeds were counted daily until the 10th day to calculate the following parameters:

(1) Germination percentage (GP in %):

$$\mathrm{GP}\;(\%)=\frac{\mathrm{Number}\;\mathrm{of}\;\mathrm{germinated}\;\mathrm{seeds}}{\mathrm{Total}\;\mathrm{number}\;\mathrm{of}\;\mathrm{sown}\;\mathrm{seeds}}\times 100$$

(2) Germination rate index (GRI) was calculated according to Esechie [75] as GRI = (G1 + G2 + … + Gi)/i, where G1 is the germination percentage, calculated daily from day 1 to i = 10. It gives an indication of the percentage of seeds germinating per day during the germination test period.
(3) Coefficient of velocity of germination (CVG) was calculated following Al-Ansari and Ksiksi [76] as follows: CVG = (N1 + N2 + … + Ni) × 100/( N1*T1 + … + Ni*Ti), where N is the number of seeds germinated every day and T is the number of days from seeding corresponding to N. It gives an indication of the speed of germination. CVG values increase when the number of germinated seeds increases and the time required for germination decreases.
(4) Germination index (GI) was calculated according to Benech Arnold et al. [77] as follows: GI = (10 × N1) + (9 × N2) + … + (1 × N10); where N1, N2, …, N10, is the number of seeds germinated on the first, second and subsequent days until the 10th day, and the multipliers (i.e., 10, 9 … etc.) are weights given to the days of the germination. It measures both percentage and speed of germination. High values indicate that seeds germinate early and low values that seeds germinate late.
(5) Mean germination time (MGT) represents the mean time that seeds require to initiate and end germination. It was calculated according to Orchard [78] as follows:
  MGT = Σ(Ti × Ni)/ΣNi
  where Ni is the number of the newly germinated seeds at time Ti.
(6) Seedling vigor index (SVI) was calculated according to Abiri et al. [79] with little modification as a multiplication of the final germination percentage by the total seedling length (shoot and root lengths).
(7) Shoot length (ShL in cm) and root length (RL in cm) were measured manually at the tenth day of germination using a scaled ruler for five seedlings from each replicate at the end of the experiment.
(8) Seedling length (SL in cm) was measured as the total of root and shoot lengths.
(9) Root–shoot ratio (RSR) was calculated as a ratio between root length and shoot length.
(10) Seedlings fresh weight (SFW in g) and seedling dry weight (SDW in mg) were recorded by weighing harvested seedlings at day 10 and, respectively, after drying the fresh weight at 80 °C for 72 h to obtain the SDW using an ultra-micro lab balance (Sartorius AC 1215, Germany).
(11) Water content percentage (WCP in %) was calculated based on the following formula:

$$\mathrm{WCP}\;(\%)=\frac{\left(\mathrm{SFW}-\mathrm{SDW}\right)}{\mathrm{SFW}}\times 100$$

### 4.4. Stress Tolerance Indices (STI)

In order to evaluate the growth performance and the variation among genotypes in their tolerance to salinity, stress tolerance indices (STI) were derived for the following eight parameters:

(1) Germination percentage (GPTI, as germination percentage tolerance index);
(2) Mean germination time (MGTTI, as mean germination time tolerance index);
(3) Shoot length (ShLTI, as shoot length tolerance index);
(4) Root length (RLTI, as root length tolerance index);
(5) Seedling length (SLTI, as seedling length tolerance index);
(6) Seedling fresh weight (SFWTI, as seedling fresh weight tolerance index);
(7) Seedling dry weight (SDWTI, as seedling dry weight tolerance index);
(8) Water content % (WCPTI, as water content percentage tolerance index).

The salt tolerance indices (STIs) for these traits were calculated according to the formula of Fernandez [80]:

$$STI=(Y_{p}\times Y_{s})/{\left({\bar{X}}_{p}\right)}^{2}$$

where *Ys* and *Yp* are the traits of interest of the tested genotypes under salinity (stress) and non-stress conditions (control), and ${\bar{X}}_{p}$ is the mean value of the trait under non-stress conditions.

### 4.5. Statistical Analyses

The separate and combined analyses of variance (ANOVA) of a completely randomized experiment were performed using SAS software v. 9.2 with PROC GLM procedure [81], to test the effect of each treatment and the interaction between the *intermedium-spike* accessions and salinity treatments. Broad-sense heritability (Hb) estimates were calculated under control and salinity conditions following Padi [82].

$$H_{b}=\frac{\sigma_{g}^{2}}{\sigma_{p}^{2}},\;\;\;\;\;\;\;\;\;\;\;\;\;\;\;\sigma_{p}^{2}=(\sigma_{g}^{2})+\left(\frac{\sigma_{e}^{2}}{r}\right)$$

where $\sigma_{g}^{2}$ is the genotypic variance, $\sigma_{p}^{2}$ is the phenotypic variance, $\sigma_{e}^{2}$ is the pooled error variance, and r is the number of replicates. Additionally, least square means (Ls-means) were calculated for each genotype using PROC GLM method of SAS software. The phenotypic Pearson correlation matrix analysis among the traits under control and 150 mM NaCl treatments was calculated by R-studio.

### 4.6. GWAS Analysis

The GWAS was conducted as previously described by Dreissig et al. [83] with the software SAS 9.4 [84] for 192 accessions with valid genotype and phenotype data. In a first step, all SNPs associated with the target trait are selected using a multiple linear regression model (SAS PROC GLMSELECT). Then, 100 repeated subsamples are created with 80% of the accessions, and only those SNPs that improve the prediction of the remaining 20% are selected (according to the minimum average squared error), and all SNPs selected more than once in this step are considered potential cofactors. The potential cofactors were used as input for the final cofactor selection (SAS PROC GLMSELECT based on the Schwarz Bayesian Criterion) in the whole dataset. The selected co-factors are then modeled with SAS PROC REG in the background of a multiple linear regression model where all SNPs are tested for significance. Thus, allele effects, R² and *p* value are estimated as a function of the cofactors, which enter the model first according to their ranking in the previous step, by applying the model option PARTIALR2 (SEQTESTS). *p* values were corrected for multiple testing based on Bonferroni [85], SNPs with Bonferroni *p* < 0.05 were defined as significant.

### 4.7. GWAS’ Significant QTL Annotation

To determine whether genes surrounding significant loci are enriched for specific GOs, the genes located within a region of ±100 kb next to the significant SNPs were selected as candidates for annotation and pathway analyses. Cluster of Orthologous Groups (COG) analysis of proteins was performed using the NCBI website (http://www.ncbi.nlm.nih.gov/COG/ (accessed on 29 June 2022)).

## Figures and Tables

**Table 1:** Mean squares (MS) of separate and combined ANOVA of salinity treatments, coefficient of variation (CV%), coefficient of determination (R^2^), and broad-sense heritability (H_b_) for the investigated traits in 208 accessions (A) under two treatments (T); control and salinity treatment (150 mM NaCl).

Trait	Control				150 mM NaCl				Combined ANOVA		
	MS (df = 207)	CV	R^2^	H_b_	MS	CV	R^2^	H_b_	T (df = 1)	A (df = 207)	A × T (df = 207)
**GP**	502.1 **	6.5	0.89	93.6	980.8 **	16.3	0.75	83.8	33,750.1 **	1177.7 **	305.2 **
**GRI**	33,928 **	8.8	0.94	96.9	31,024 **	18.1	0.84	90.6	1,659,664 **	54,132 **	10,821 **
**CVG**	123.5 **	10.4	0.85	90.9	54.5 **	9.1	0.80	87.7	4496.0 **	135.6 **	42.4 **
**GI**	2.5 **	7.8	0.95	97.6	2.1 **	18.5	0.84	90.2	163.25 **	3.87 **	0.75 **
**MGT**	1.5 **	9.4	0.89	93.9	0.9 **	9.5	0.78	86.3	40.0 **	1.9 **	0.6 **
**SVI**	677,900 **	12.8	0.85	91.5	531,966 **	19.4	0.86	91.6	195,406,315 **	958,912 **	250,954 **
**ShL**	12.8 **	11.5	0.82	89.2	15.7 **	13.6	0.91	95.1	4406.6 **	19.7 **	8.8 **
**RL**	14.2 **	12.3	0.79	87.0	10.7 **	11.6	0.88	93.2	4377.8 **	17.7 **	7.1 **
**SL**	44.6 **	10.7	0.81	88.4	47.4 **	11.2	0.91	95.0	17,569 **	64.6 **	27.3 **
**RSR**	0.1 **	11.1	0.79	86.4	0.4 **	14.0	0.87	92.8	5.4 **	0.3 **	0.2 **
**SFW**	10,748 **	13.1	0.79	86.5	7743 **	24.8	0.61	67.8	2,484,890 **	13,005 **	5486 **
**SDW**	161.0 **	13.9	0.82	89.2	221.5 **	22.3	0.61	68.5	17,361.2 **	295.4 **	87.1 **
**WCP**	6.8 **	1.1	0.78	85.7	38.2 **	3.1	0.76	84.1	24,654.2 **	24.8 **	20.3 **

Variable abbreviations are as follows: GP: germination percentage, GRI: germination rate index (days), CVG: coefficient of velocity of germination, GI: germination index, MGT: mean germination time (days), SVI: seedling vigor index, ShL: shoot length (cm), RL: root length (cm), SL: seedling length (cm), RSR: root: shoot ratio, SFW: seedling fresh weight (mg), SDW: seedling dry weight (mg), WCP: water content percentage (%). ** highly significant at 0.01 probability level.

**Table 2:** Means, summary statistics, and relative salinity performance (R%) due to salinity effects on the studied traits as compared to control.

Trait	Control				Salinity				R%
	Mean	Std Error	Minimum	Maximum	Mean	Std Error	Minimum	Maximum	
**GP**	87.77	0.55	30.0	100.0	77.37	0.83	10.00	100.00	−11.85
**GRI**	370.89	4.38	77.8	555.6	297.95	4.43	11.11	511.11	−19.66
**CVG**	32.13	0.28	17.5	50.0	28.33	0.19	16.67	47.37	−11.82
**GI**	3.17	0.04	0.7	5.0	2.45	0.04	0.17	4.67	−22.81
**MGT**	3.27	0.03	2.0	5.7	3.63	0.02	2.11	6.00	10.95
**SVI**	1880.99	20.56	644.0	3306.7	1089.60	18.19	11.00	2483.33	−42.07
**ShL**	10.23	0.09	4.1	17.5	6.47	0.10	0.20	13.00	−36.75
**RL**	11.10	0.10	4.0	18.0	7.35	0.08	0.60	14.37	−33.75
**SL**	21.33	0.17	8.3	35.5	13.82	0.17	0.80	24.83	−35.19
**RSR**	1.11	0.01	0.6	1.8	1.24	0.02	0.58	5.00	11.94
**SFW**	291.00	2.70	159.2	554.9	201.76	2.61	30.70	609.60	−30.67
**SDW**	30.01	0.32	14.1	64.4	37.47	0.44	10.00	123.20	24.86
**WCP**	89.63	0.07	83.2	93.0	80.74	0.16	51.12	93.46	−9.92

Variable abbreviations are as follows: GP: germination percentage, GRI: germination rate index (days), CVG: coefficient of velocity of germination, GI: germination index, MGT: mean germination time (days), SVI: seedling vigor index, ShL: shoot length (cm), RL: root length (cm), SL: seedling length (cm), RSR: root: shoot ratio, SFW: seedling fresh weight (mg), SDW: seedling dry weight (mg), WCP: water content percentage (%).

**Table 3:** Mean squares (MS) of accessions, coefficient of variation (CV%), coefficient of determination (R^2^), broad-sense heritability (H_b_), and descriptive statistics for the salinity tolerance indices.

Index	MS (df = 207)	CV	R^2^	H_b_	Means	Std Error	Minimum	Maximum
**GPTI**	0.24 **	17.3	0.83	89.9	0.90	0.012	0.06	1.30
**MGTTI**	0.23 **	10.8	0.92	95.9	0.90	0.012	0.27	1.91
**ShLTI**	0.29 **	18.9	0.90	94.7	0.65	0.013	0.03	1.78
**RLTI**	0.21 **	17.3	0.88	93.5	0.68	0.011	0.06	1.81
**SLTI**	0.21 **	16.0	0.90	94.7	0.66	0.011	0.05	1.65
**SFWTI**	0.24 **	26.4	0.77	85.2	0.71	0.013	0.11	2.10
**SDWTI**	1.01 **	26.9	0.81	88.1	1.29	0.026	0.28	5.08
**WCTI**	0.01 **	3.3	0.78	85.6	0.90	0.002	0.59	1.02

GPTI: germination tolerance index, MGTTI: mean germination time tolerance index, SHLTI: shoot length tolerance index, RLTI: root length tolerance index, SLTI: seedling length tolerance index, SFWTI: seedling fresh weight tolerance index, SDWTI: seedling dry weight tolerance index, WCTI: water content tolerance index. Two asterisks (**) indicate highly significant mean squares of line effects at 0.01 probability level.

**Table 4:** Putative QTL associated with germination and seedling growth related traits under control and salinity conditions as well as salinity tolerance indices.

Trait	Treatment	Marker	Chr	Position bp	Raw_*p*	Log10_Raw_*p*	Estimate
**QTL detected under control treatment**							
**GP**	Control	6_7696839	6H	7,696,839	1.64 × 10^−6^	5.79	8.98
**CVG**	Control	1_546649042	1H	546,649,042	2.07 × 10^−7^	6.68	6.86
**GI**	Control	1_546649042	1H	546,649,042	6.68 × 10^−7^	6.18	0.94
**MGT**	Control	2_708864195	2H	708,864,195	9.67 × 10^−7^	6.01	0.5
**SFW**	Control	7_14906512	7H	14,906,512	6.61 × 10^−9^	8.18	52.82
**SDW**	Control	7_49898546	7H	49,898,546	4.90 × 10^−10^	9.31	9.28
**QTL detected under salinity treatment**							
**GP**	Salinity	5_586496095	5H	586,496,095	1.43 × 10^−6^	5.84	−13.86
**CVG**	Salinity	1_546373322	1H	546,373,322	3.76 × 10^−6^	5.42	4.58
**GI**	Salinity	1_546373322	1H	546,373,322	1.02 × 10^−6^	5.99	0.94
**SVI**	Salinity	5_3766117	5H	3,766,117	8.43 × 10^−8^	7.07	353.02
**ShL**	Salinity	2_151892989	2H	151,892,989	1.10 × 10^−6^	5.96	2.66
**ShL**	Salinity	5_560970990	5H	560,970,990	2.27 × 10^−7^	6.64	−2.66
**RL**	Salinity	2_420745590	2H	420,745,590	8.71 × 10^−8^	7.06	−1.68
**SL**	Salinity	5_561126360	5H	561,126,360	2.58 × 10^−7^	6.59	−5.12
**SL**	Salinity	7_637877064	7H	637,877,064	2.22 × 10^−7^	6.65	3.04
**SFW**	Salinity	1_487884122	1H	487,884,122	3.79 × 10^−11^	10.42	−63.42
**SFW**	Salinity	3_599466174	3H	599,466,174	1.49 × 10^−6^	5.83	−42.16
**SDW**	Salinity	1_15066059	1H	15,066,059	5.23 × 10^−8^	7.28	8.84
**WCP**	Salinity	7_629887676	7H	629,887,676	2.47 × 10^−6^	5.61	−2.86
**QTL detected for salinity tolerance indices**							
**ShLTI**	Index	2_420745590	2H	420,745,590	2.25 × 10^−10^	9.65	−22.08
**RLTI**	Index	2_698868743	2H	698,868,743	4.14 × 10^−8^	7.38	−15.96
**SLTI**	Index	2_420745590	2H	420,745,590	1.48 × 10^−9^	8.83	−17.58
**SFWTI**	Index	7_70461482	7H	70,461,482	6.25 × 10^−7^	6.20	−14.92

Variable abbreviations are as follows: GP: germination percentage, GRI: germination rate index (days), CVG: coefficient of velocity of germination, GI: germination index, MGT: mean germination time (days), SVI: seedling vigor index, ShL: shoot length (cm), RL: root length (cm), SL: seedling length (cm), RSR: root: shoot ratio, SFW: seedling fresh weight (mg), SDW: seedling dry weight (mg), and WCP: water content percentage (%), SHLTI: shoot length tolerance index, RLTI: root length tolerance index, SLTI: seedling length tolerance index, SFWTI: seedling fresh weight tolerance index.

**Table 5:** Putative HC candidate genes associated with identified QTL related to control and salinity treatments.

Marker	Candidate Gene	Chr.	Start	End	Function Description
m1_15066059	HORVU1Hr1G007420	1H	15,064,891	15,064,891	Cytochrome P450 superfamily protein
m1_466766698	HORVU1Hr1G065250	1H	466,760,732	466,760,732	Potassium channel AKT2
m1_487884122	HORVU1Hr1G069990	1H	487,882,055	487,882,055	U-box domain-containing protein 16
m1_528149209	HORVU1Hr1G081810	1H	528,147,129	528,147,129	26S proteasome non-ATPase regulatory subunit 1 homolog A
m1_546373322	HORVU1Hr1G090570HORVU1Hr1G090580	1H1H	546,411,012546,421,876	546,412,822546,423,244	Phospholipase A1-II 6
m2_419658044	HORVU2Hr1G062320	2H	419,657,547	419,657,547	tRNA dimethylallyltransferase 2
m2_698868743	HORVU2Hr1G102970	2H	698,868,231	698,868,231	F-box/RNI-like superfamily protein
m2_710957942	HORVU2Hr1G106970	2H	710,953,055	710,953,055	Homeobox-leucine zipper protein family
m3_166063769	HORVU3Hr1G032440	3H	166,062,751	166,062,751	Two-component response regulator-like APRR2
m3_361778117	HORVU3Hr1G050400	3H	361,773,216	361,773,216	Peptidyl-prolyl cis-trans isomerase-like 1
m3_599466174	HORVU3Hr1G082580	3H	599,464,312	599,464,312	Protein NRT1/ PTR FAMILY 5.10
m3_603625697	HORVU3Hr1G083460	3H	603,625,804	603,625,804	DOF zinc finger protein 1
m3_648172294	HORVU3Hr1G094870	3H	648,170,663	648,170,663	histidine kinase 3
m3_662456881	HORVU3Hr1G099770	3H	662,455,277	662,455,277	hydroxyethylthiazole kinase family protein
m4_631432489	HORVU4Hr1G085320	4H	631,514,701	631,519,979	NAC domain protein
m4_608376198	HORVU4Hr1G078670	4H	608,370,056	608,370,056	Mitochondrial substrate carrier family protein
m5_3766117	HORVU5Hr1G001090	5H	3,762,210	3,762,210	BEL1-like homeodomain 6
m5_659835561	HORVU5Hr1G121610	5H	659,835,141	659,835,141	B-cell receptor-associated protein 31-like
m6_7696839	HORVU6Hr1G003300	6H	7,696,550	7,696,550	nitrate reductase 1
m6_32557121	HORVU6Hr1G014910	6H	32,555,240	32,555,240	D.melanogaster polytene
m7_49898546	HORVU7Hr1G027930	7H	49,898,118	49,898,118	Raffinose synthase family protein
m7_70461482	HORVU7Hr1G034050	7H	70,456,816	70,456,816	Pentatricopeptide repeat-containing protein
m7_161050366	HORVU7Hr1G047740	7H	161,049,541	161,049,541	L-gulonolactone oxidase 2
m7_167285781	HORVU7Hr1G048720	7H	167,284,758	167,284,758	Heme A synthase

## Data Availability

Not applicable.

## Supplementary Materials

The supporting information can be downloaded at: https://www.mdpi.com/article/10.3390/ijms231911060/s1.
